# Phylogenomics, evolution and origin of multidrug-resistant Acinetobacter baumannii ST15

**DOI:** 10.1099/mgen.0.001450

**Published:** 2025-07-17

**Authors:** Eradah Abu Sabah, Liam A. Tobin, Francois Lebreton, Patrick T. McGann, Mehrad Hamidian

**Affiliations:** 1 Australian Institute for Microbiology and Infection, University of Technology Sydney, Ultimo, NSW, Australia; 2 Multidrug-Resistant Organism Repository and Surveillance Network (MRSN), Bacterial Diseases Branch, CIDR , Walter Reed Army Institute of Research, Silver Spring, MD, USA

**Keywords:** *Acinetobacter baumannii*, antibiotic resistance, ST15, ST3, plasmid, homologous recombination

## Abstract

We studied the genomic evolution and transmission dynamics of multidrug-resistant ST15 (Institut Pasteur scheme) *Acinetobacter baumannii*, examining resistance gene acquisition, clonal diversification, geographic distribution and origin of this high-risk clone. One hundred and fifty-two (*n*=152) ST15 genomes from 18 countries (1997–2024), including 42 isolates from U.S. Military Treatment Facilities and 110 publicly available genomes in GenBank, were analysed. Whole-genome sequencing, assembly and annotation were performed using established bioinformatics pipelines. Phylogenetic analysis based on core-genome SNPs – filtered for recombination with *Gubbins* – was combined with mobile element and resistance gene identification. ST15 isolates separated into two main clades with distinct subclades and variable resistance profiles. Homologous recombination drove the diversification of resistance determinants, including multiple *ampC* alleles. Key resistance genes, such as *bla*_OXA-23_, were disseminated via known transposons (Tn*2006* or Tn*2008*), while plasmid exchange, including *dif* module acquisitions, also played a role in the spread of *bla*_CARB_. Patristic analysis identified Argentina as the likely origin for the emergence of ST15, aligning with early 1997 isolates. Recombination, transposon-mediated gene transfer and plasmid exchange have been central in driving the evolution and global dissemination of ST15.

**Impact Statement**

*Acinetobacter baumannii* is a critical Gram-negative pathogen due to its high incidence of globally distributed antibiotic-resistant clones. This comprehensive genomic analysis of the ST15 multidrug-resistant clone reveals the complex evolutionary mechanisms underpinning the emergence and global dissemination of this clone. This study shows the genetic diversity, phylogenetic relationships and resistance gene acquisition, highlighting the important role of homologous recombination and mobile genetic elements in the diversification and antibiotic resistance gene acquisition of ST15. The identification of distinct clades and the tracing of transmission pathways also reveal a complex evolutionary history, with Argentina suggested as a potential origin. These findings advance the understanding of antibiotic resistance evolution in *A. baumannii* and underscore the necessity for ongoing genomic surveillance to identify potential sources and mitigate the spread of resistant strains.

## Data Summary

*Acinetobacter baumannii* genomes sequenced in this study, including draft and complete genomes, are deposited in GenBank and available under the BioProject PRJNA1199583. All publicly available *A. baumannii* genomes (*n*=110) used in this study were downloaded from GenBank with their accession numbers listed in Table S1.

## Introduction

Antibiotic resistance is a significant public health threat and a growing global concern [1]. *Acinetobacter baumannii* is a particularly concerning pathogen due to its high levels of resistance against multiple antibiotics, including those considered last resort such as carbapenems, in outbreak strains [2]. The primary resistance mechanism in *A. baumannii* is the acquisition of antibiotic resistance genes (ARGs) via mobile genetic elements, including genomic islands, plasmids and transposons [1,4]. Additionally, clonal expansion has played a major role in the global dissemination of resistant *A. baumannii* clones, with most outbreak strains often belonging to a limited number of sequence types (STs), including ST1, ST2, ST10, ST15, ST25, ST79 and ST85. While ST1 and ST2 (also referred to as global clones 1 and 2 or international clones 1 and 2, respectively) have been extensively studied [5], other resistant clones, like ST15 (also reported as International Clone 4 or IC4), remain under-investigated, requiring further analysis to uncover their genetic profiles, resistance mechanisms, origin and genomic evolution.

ST15 (Institute Pasteur scheme) strains have been reported globally in clinical samples in countries such as Brazil, Ecuador, Chile, Paraguay, Spain, Greece, Iran, Turkey and Egypt [6,13]. These strains were mainly associated with carbapenem resistance due to *bla*_OXA-23_ and *bla*_OXA-58_, particularly those recovered in South American countries [11,12, 14].

Military conflicts often contribute to the rise and spread of multidrug-resistant (MDR) micro-organisms, including *A. baumannii* [15]. Here, we analysed 42 ST15 genomes from the Multidrug-Resistant Organism Repository and Surveillance Network (MRSN) project. These genomes were recovered from the U.S. Military Treatment Facilities and originated primarily from Middle Eastern battle zones in Iraq, in addition to one strain from Afghanistan and one from Ukraine. To put these genomes into a global perspective, we compared them with an additional 110 ST15 genomes (including 10 single locus variants of ST15) from GenBank, resulting in a total analysis of 152 MDR *A. baumannii* genomes. Our results reveal that ST15 strains are present in diverse geographical regions worldwide, with South America identified as the probable origin of this clonal lineage. Additionally, we provide evidence that homologous recombination plays a significant role in the diversification of ST15 strains, enabling the acquisition of resistance genes and other adaptive traits including genomic loci encoding capsular polysaccharides.

## Methods

### Genomes studied

A total of 152 genomes (including 142 ST15 and 10 single locus variants of ST15, i.e. ST84, ST238, ST318, ST1038 and ST1447) were analysed in this study, including 42 genomes from the MRSN project sequenced in this study (Table S1, available in the online Supplementary Material). The MRSN strains were recovered in U.S. Military Treatment Facilities in the USA and Baghdad, Iraq, between 2003 and 2011. Additionally, 110 ST15 genomes were identified in GenBank and included in this study.

### Whole genome sequencing, genome assembly and sequence analysis

The genome sequences of the *n*=42 MRSN strains were determined on an Illumina MiSeq platform, producing 150 bp paired-end reads. Quality control of the raw sequencing data was performed using *FastQC* [16]. Adapter sequences were identified and removed using the *Btrim* v.0.2.0 program [17]. Short reads were *de novo* assembled into draft genomes using *Newbler* (https://github.com/etheleon/newbler) for all genomes determined here, as well as 17 strains for which only short-read data were available in National Center for Biotechnology Information (NCBI). In addition, the genome sequence of five (*n*=5) representative strains was completed using the combination of short- and long-read (MinION) sequencing technologies (Table 1). Long-read sequencing was done using the R10.4.1 flow cell. Basecalling was conducted with *Dorado* v9.0.0 (https://github.com/nanoporetech/dorado/) using the super-accurate model (dna_r10.4.1_e8.2_400bps_sup@v5.0.0). *De novo* genome assembly was carried out using *Autocycler* v0.1.2 (https://github.com/rrwick/Autocycler/tree/v0.2.0). The resulting assemblies were further polished using Illumina short-read data with *Polypolish* (https://github.com/rrwick/polypolish).

**Table 1.** Properties of complete ST15 genomes

Strain	Size (bp)	PlasmidRep	Mob	Acquired resistancegene	Important plasmid-encoded function	GenBank no.
**MRSN15091**	4,010,550	NA*		*aphA*6, *bla_O_*_XA-23_		**CP179883**
p1MRSN15091	6,359	R3-T13	–	–	Toxin and antitoxin, enhances zinc uptake during infection	CP179884
p2MRSN15091	10,848	R3-T14	Mob_Q_	–	Toxin and antitoxin, putative TonB-dependent receptor	CP179885
p3MRSN15091	76,810	R3-T3	–	*sul2*	DNA replication and repair, stress response proteins	CP179886
**MRSN548254**	3,943,904	na		*sul*2		**CP179880**
p1MRSN548254	6,078	–	Mob_P_	*aadB*		CP179881
p2MRSN548254	13,932	R3-T14	Mob_Q_	*bla*_CARB-50_	Toxin and antitoxin, stress response (USP)	CP179882
**MRSN548102**	3,946,353	na		*sul*2		**CP179875**
p1MRSN548102	6,078	–	Mob_P_	*aadB*		CP179876
p2MRSN548102	6,202	R3-T10	–	*tet39*		CP179877
p3MRSN548102	11,196	R3-T17	–		Toxin and antitoxin, nutrient transport; stress response regulation	CP179878
p4MRSN548102	13,932	R3-T14	Mob_Q_	*bla*_CARB-50_	Toxin and antitoxin, anion transport; stress response (USP)	CP179879
**MRSN15116**	4,028,264	na		*aphA*6, *bla*_OXA-23_		**CP179872**
p1 MRSN15116	13,776	R3-T14	Mob_Q_	*bla*_TEM-1_, *aacC2e*	Toxin and antitoxin	CP179873
p2MRSN15116	177,375	–		*sul2*, *strAB*	Toxin and antitoxin, mercury resistance; stress response and DNA repair; metal ion transport	CP179874
**MRSN122172**	4,003,826	na		*aphA6*		**CP179869**
p1MRSN122172	10,848	R3-T14	Mob_Q_	–	Toxin and antitoxin, nutrient transport via the TonB system	CP179870
p2MRSN122172	162,333	–		*strAB*, *sul2*, *floR*, *mph-msr*(E)	Toxin and antitoxin, phage defence via BREX system; transcriptional regulation of streptomycin and mercury resistance; stress response and DNA repair	CP179871

*Not applicable.

Genome annotation was performed using *Prokka* v1.13.4 [18]. Mobile genetic elements were identified with tools such as *ISFinder* [19] and Standalone blast v2.16.0 [20]. Antibiotic resistance genes were identified using the *Abricate* v0.8.13 software [21] and the *ResFinder* v4.6.0 [22] and *CARD* databases [23]. Protein-coding regions were characterized through *blastp* v2.16.0 [20] and UniProt searches [24].

STs were assigned using the Institut Pasteur and Oxford multilocus sequence typing (MLST) schemes via the *mlst* v2.16.1 program [25]. The capsular polysaccharide (KL) and lipo-oligosaccharide outer core (OCL) synthesis loci were identified using *Kaptive* v.3.0 [26]. The *CheckM* v1.2.3 software (available at https://github.com/Ecogenomics/CheckM) was used to examine the quality (i.e. completeness and contamination) of all genomes studied here, including those we found in NCBI and also the genomes we sequenced and assembled in this study [27].

Chromosomal regions acquired by homologous recombination were identified using *Gubbins* [28], and the results were visualized in *R* using the *plotTree* v.1.0 package (https://github.com/katholt/plotTree).

Plasmid replication initiation genes (*rep*/Rep types) were determined using the *Acinetobacter* plasmid typing (APT) scheme [29]. Manual annotation of regions of interest was conducted using *SnapGene^®^* v6.0.5 software, and figures were drawn to scale using *Illustrator^®^* v26.2.1. Prophage genomes were detected in representatives of each clade using the *PHASTEST* (PHAge Search Tool with Enhanced Sequence Translation) program (available at https://phastest.ca/) [30].

### Phylogenetic analysis

Maximum likelihood (ML) phylogenetic trees were initially constructed using Snippy-aligned sequences and analysed with *IQ-TREE* v2.3.6. The high-quality core-genome SNPs identified were used for further refinement. *Gubbins* v2.4.1 was then applied to the output of *Panaroo* v1.5.1 (https://github.com/gtonkinhill/panaroo) to identify recombinant regions and refine the phylogenetic analysis by excluding horizontally transferred sequences (via recombination). We inferred the final ML phylogenetic tree from the alignment of substitution mutations (SNPs not associated with recombination events), rooted using a GC1 outgroup (i.e. *A. baumannii* A1; GenBank no. CP010781). The resulting phylogenetic tree was visualized using *FigTree* v1.4.4. Additionally, antimicrobial resistance genes and the phylogenetic tree were plotted against the recombination-free tree using the *ggplot2* [31] v3.5.1 and *plotTree* (https://github.com/katholt/plotTree) packages in *R*.

Patristic distances, representing evolutionary distances between strains, were used to investigate the origins of the ST15 genomes studied here. Patristic distances were calculated using the *PATRISTIC* program [32]. The distance matrix was then sorted to identify the closest strain pairs, excluding self-pairings, allowing for analysis of strain transfer and spread across different regions. Results were visualized in *R* using *ggplot2* v3.5.1 [31].

## Results and discussion

### General properties, geographical distribution and phylogenetic relationships

**Fig. 1.**
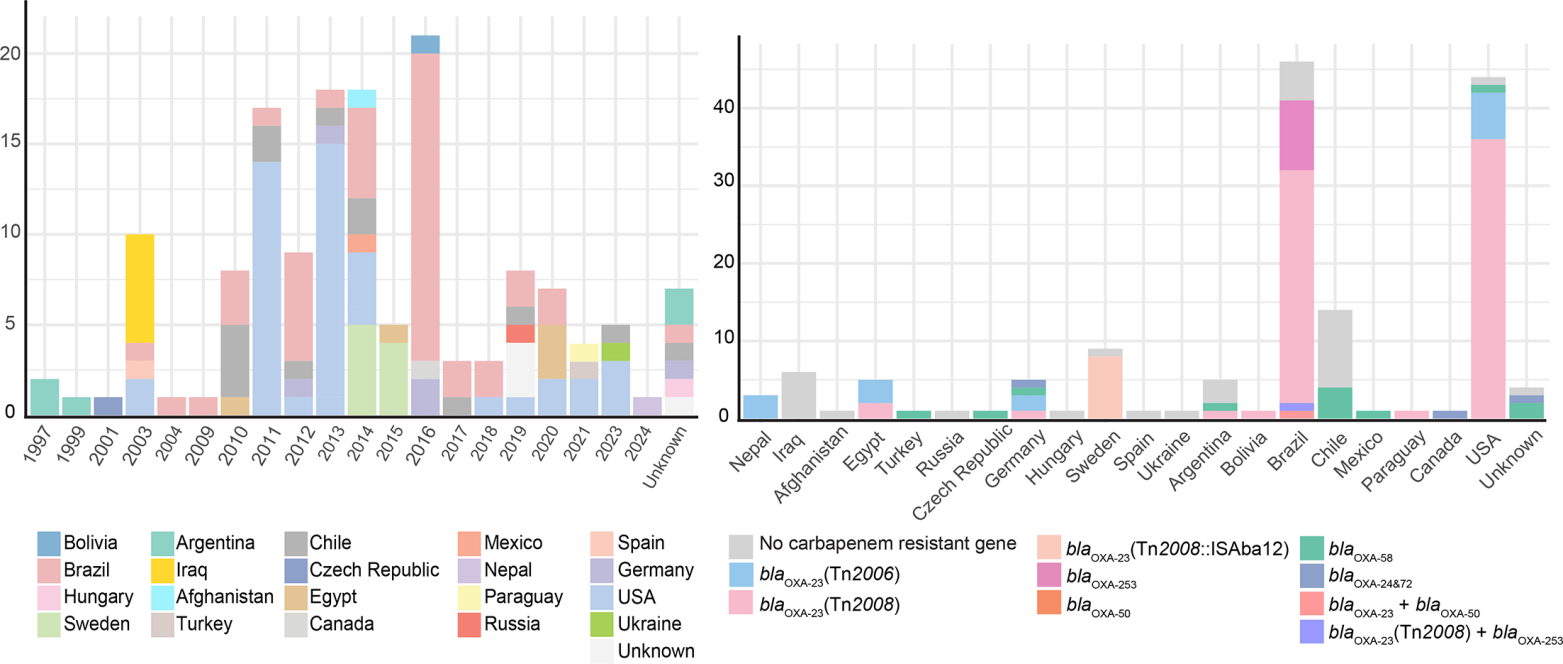
Stacked bar plots indicating the isolation dates of ST15 genomes (left) and distribution of carbapenem resistance genes in each country of origin (right). Individual carbapenem resistance genes and countries of isolation are colour-coded and shown in the key sections.

We studied a total of 152 genomes belonging to the ST15 clonal complex (142 ST15 genomes and 10 belonging to ST15, i.e. ST84, ST238, ST318, ST1038 and ST1447) (Table S1) . Amongst these, 42 genomes belong to the MRSN collection and were sequenced in this study. The remaining 110 genomes were found in GenBank. The other GenBank genomes were from an additional 16 different countries, including those in South America, North America, Europe, Africa and Asia (Fig. 1). These strains were from a diverse timespan, ranging from 1997 to 2024, with 2016 including the largest number of genomes (*n*=21), followed by 2014, 2013 and 2011 (Fig. 1). Overall, the highest number of genomes were from Brazil (*n*=46), followed by the USA (*n*=44) (Fig. 1).

**Fig. 2.**
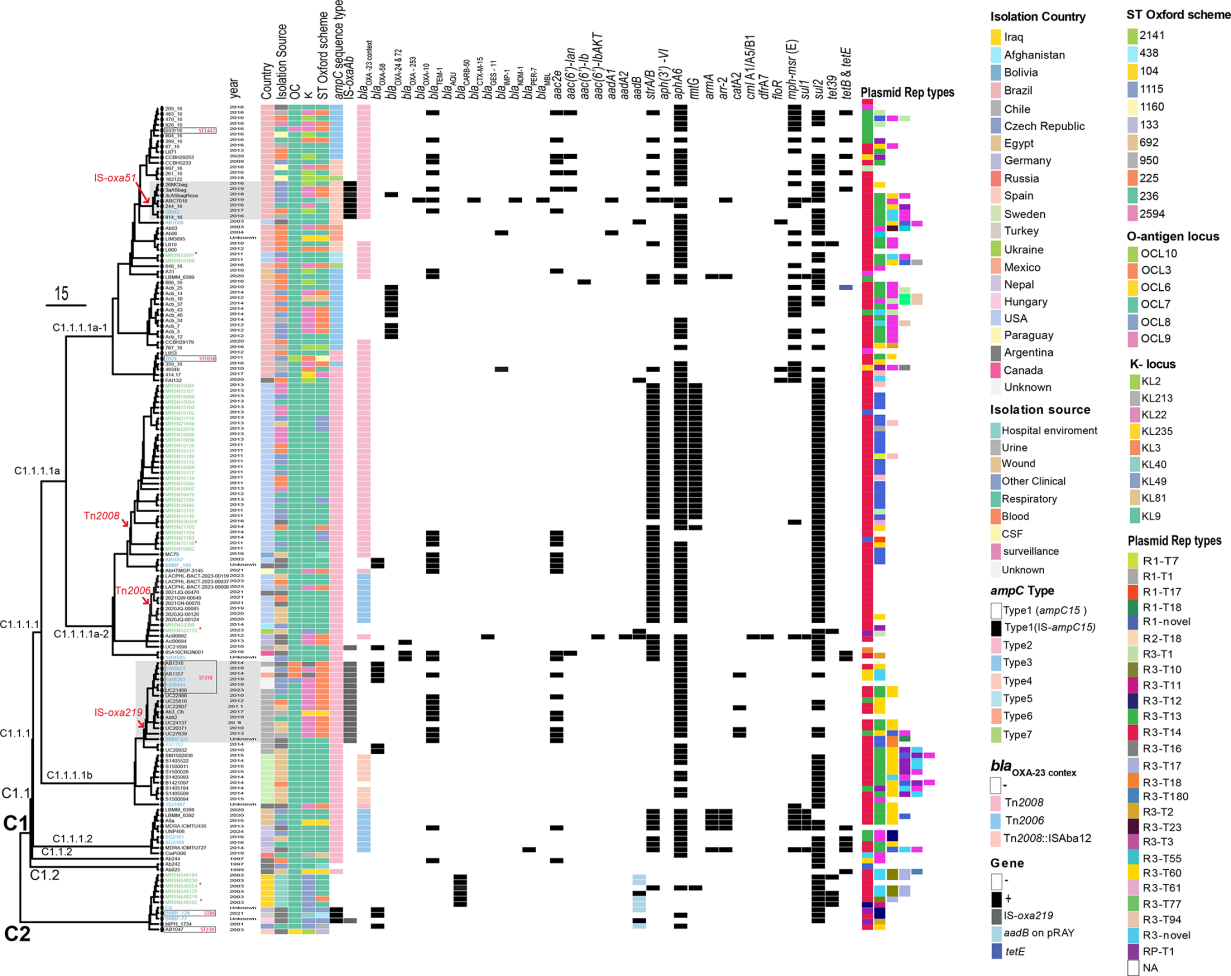
Phylogenetic tree depicting genetic relationships amongst ST15 *A. baumannii* isolates. Metadata is aligned to the right of the tree, corresponding to each strain tip. ‘NK’ denotes data that are not publicly available. Red arrows indicate insertion events of resistance genes, including *bla*_OXA-23_ via Tn*2006* or Tn*2008*. Strains marked in green were sequenced and analysed as part of this study. Strains marked in red (*) represent completed genomes from this study. Those in blue are genomes for which we found short-read data in NCBI and assembled in this study. ST15 SLV genomes are boxed with their STs indicated inside the box.

In *A. baumannii*, surface polysaccharide (CPS) loci genes are clustered at the K locus (KL, capsule locus) and OC locus (OCL, outer core (of lipo-oligosaccharide) locus). Except for seven genomes (with OCL3, OCL6, OCL8 and OCL10), all other genomes in this study (*n*=145) carried OCL7. However, we observed more diversity in the K locus with 9 K types observed across the set (KL2, KL213, KL22, KL23, KL235, KL3, KL40, KL49, KL81 and KL9) (Fig. 2 and Table S1). The most predominant capsule locus profiles were KL9 (*n*=84), followed by KL22 (*n*=33), KL2 (*n*=12) and KL49 (*n*=10). Given these diversities and that the *gpi* gene (one of the genes used in the Oxford scheme ST) is within the K locus, ST15 genomes belonged to various Oxford STs, including ST236, ST104, ST225, ST2594, ST2141, ST692, ST1160, ST950, ST1115, ST133 and ST438 (Table S1 and Fig. 2).

Our phylogenetic analysis revealed numerous closely related strains forming distinct phylogroups; yet, a high degree of diversity was observed within each phylogroup’s accessory genomes. ST15 genomes fell into two main clades, C1 and C2 (Fig. 2). The main clade, C1, included 141 genomes, while C2 contained only 11 genomes, including 6 Iraqi genomes. The remaining five C2 genomes were from Germany, Chile, the Czech Republic, Hungary and Spain, indicating a diverse geographical spread across multiple continents.

The C1 clade is composed of two major subclades, C1.1 and C1.2. Subclade C1.2 includes only two genomes, Ab825 and Ab242 (Fig. 2), which represent the earliest ST15 strains that were publicly available. These strains, previously described as clonal MDR carbapenem-resistant strains, were recovered from patients hospitalized in the Hospital de Emergencias Clemente Alvarez (HECA) of Rosario, Argentina [33]. Our analysis indicates that they belong to a distinct novel lineage as they do not cluster with any other strain (Fig. 2).

Subclade C1.1 further splits into two main subclades. C1.1.2 includes a single genome, Ab244 (Fig. 2), representing a carbapenem-susceptible strain recovered in 1997 from the same Argentinian hospital as Ab825 and Ab242 [33]. Subclade C1.1.1 is divided into C1.1.1.1 and C1.1.1.2. The latter includes nine more recent genomes (recovered between 2013–2024) from unrelated countries, including Egypt, Nepal, Germany and Russia.

A substantial number of genomes (85%, *n*=129) fell under clade C1.1.1.1, which also divides into two major sub-clades C1.1.1.1a and C1.1.1.1b, which include 101 and 28 genomes, respectively. In fact, C1.1.1.1a represented the main ST15 clade and included all strains from Brazil, and the MRSN strains recovered in the USA (Iraqi MRSN isolates all cluster in C2).

We selected a representative genome from each of the seven major ST15 sub-clades (strains AB1047, Ab242, Ab244, JUNP406, UC22807, MRSN151091 and MRSN122172 representing C2, C1.2, C1.1.2, C1.1.1.2, C.1.1.1.1b, C1.1.1.1a-1 and C.1.1.1.1a-2, respectively) and found that each contained between one (*n*=1) and four (*n*=4) intact prophage regions.

### Distribution of carbapenem resistance genes

One hundred and thirty-six genomes (*n*=136) encoded the *bla*_OXA-51_ allele of the intrinsic *oxaAb* gene. In fact, *bla*_OXA-51_ is the first allele of the intrinsic *oxaAb* gene that was identified 20 years ago in a set of strains recovered in Argentina [34]. Seven (*n*=7) genomes contained a copy of ISAba1 upstream of *bla*_OXA-51_. All these seven genomes also included a copy of the *bla*_OXA-23_ carbapenem resistance genes in Tn*2008* – a common transposon that spreads *bla*_OXA-23_ across different sequence types and clones [5]. Seventeen strains (*n*=17) contain the *bla*_OXA-219_ allele, which differs from *bla*_OXA-51_ by a single SNP resulting in an L167V amino acid substitution in the OXA-219 protein sequence. All genomes with the *bla*_OXA-219_ allele contain a copy of ISAba1 upstream of their intrinsic *oxaAb* gene, likely enhancing the expression [35]. It appears that ISAba1 has been inserted upstream of *oxaAb* on multiple occasions given the presence of IS (next to the start codon of the *oxaAb* gene) in unrelated phylogroups (Fig. 2).

Overall, the *bla*_OXA-23_ carbapenem resistance gene, found in 104 genomes, was the most common acquired resistance gene [5]. We analysed the genomic context of *bla*_OXA-23_ and identified 14 genomes encoding bla_OXA-23_ in Tn*2006* – a class one transposon bounded by two ISAba1 copies [5]. Genomes with Tn*2006* were in C1.1.1.1a and b (Fig. 2). In the remaining genomes (*n*=82), *bla*_OXA-23_ was either in Tn*2008* (all in C1.1.1.1a) or its variant, Tn*2008*::ISAba12 (*n*=8) (all in C1.1.1.1b). Tn*2006*, Tn*2008* and Tn*2008*::ISAba12 were found in different phylogroups (Fig. 2) and in different chromosomal locations (data not shown), indicating their acquisition at multiple occasions. No specific *bla*_OXA-23_ distribution pattern was found given its presence in diverse geographical areas and phylogroups (Fig. 2). However, we noted that the majority of the MRSN genomes and those from Brazil contained a copy of *bla*_OXA-23_. Tn*2006* and Tn*2008* are the two greatest contributing transposons to the dissemination of *bla*_OXA-23_ in the two major globally distributed *A. baumannii* clones, represented by ST1 and ST2 [5]. It appears that, like other major clonal types, *bla*_OXA-23_ has also been acquired via Tn*2006* and Tn*2008* in ST15s. Other acquired carbapenem resistance genes were also found, including *bla*_OXA-58_ (in 12 genomes), *bla*_OXA-253_ (10 genomes) and *bla*_OXA-72_ (3 genomes). Other carbapenem resistance genes or those encoding extended spectrum beta-lactamases were sporadic across the set (Fig. 2).

### Other antibiotic resistance genes

Other common antibiotic resistance genes were *aphA6* (found in 117 genomes), amikacin and *sul2* (in 123 genomes) sulfonamide resistance genes. In all cases, the *aphA6* gene was in Tn*aphA6*, which is a composite (class 1) transposon bounded by ISAba125 [36]. Tn*aphA6* is the most common mobile element that spreads *aphA6,* and here, we found it inserted in different genomic contexts, including in chromosomes and plasmids (e.g. plasmids encoding the RP-T1 replication initiation protein).

The *sul2* sulfonamide resistance gene was predicted to be located within variants of the GI*sul2* genomic island, which is a 15.5 kb integrative element first identified in *Enterobacter cloacae* subsp. *cloacae* type strain ATCC 13047 (isolated in 1890), *Shigella flexneri* ATCC 700930 (recovered in 1954) and *A. baumannii* ATCC 17978 (recovered in 1951) [37]. In fact, in *A. baumannii* ATCC 17978, GI*sul2*::ISAba1 is in Tn*6174* [38], a class 3 transposon located on the pAB3 plasmid. However, in two of our complete genomes MRSN548254 and MRSN548102, as well as C4 from Germany and BMBF-128 from Turkey, Tn*6174* (containing GI*sul2*::ISAba1) was found precisely in the same chromosomal location.

**Fig. 3.**
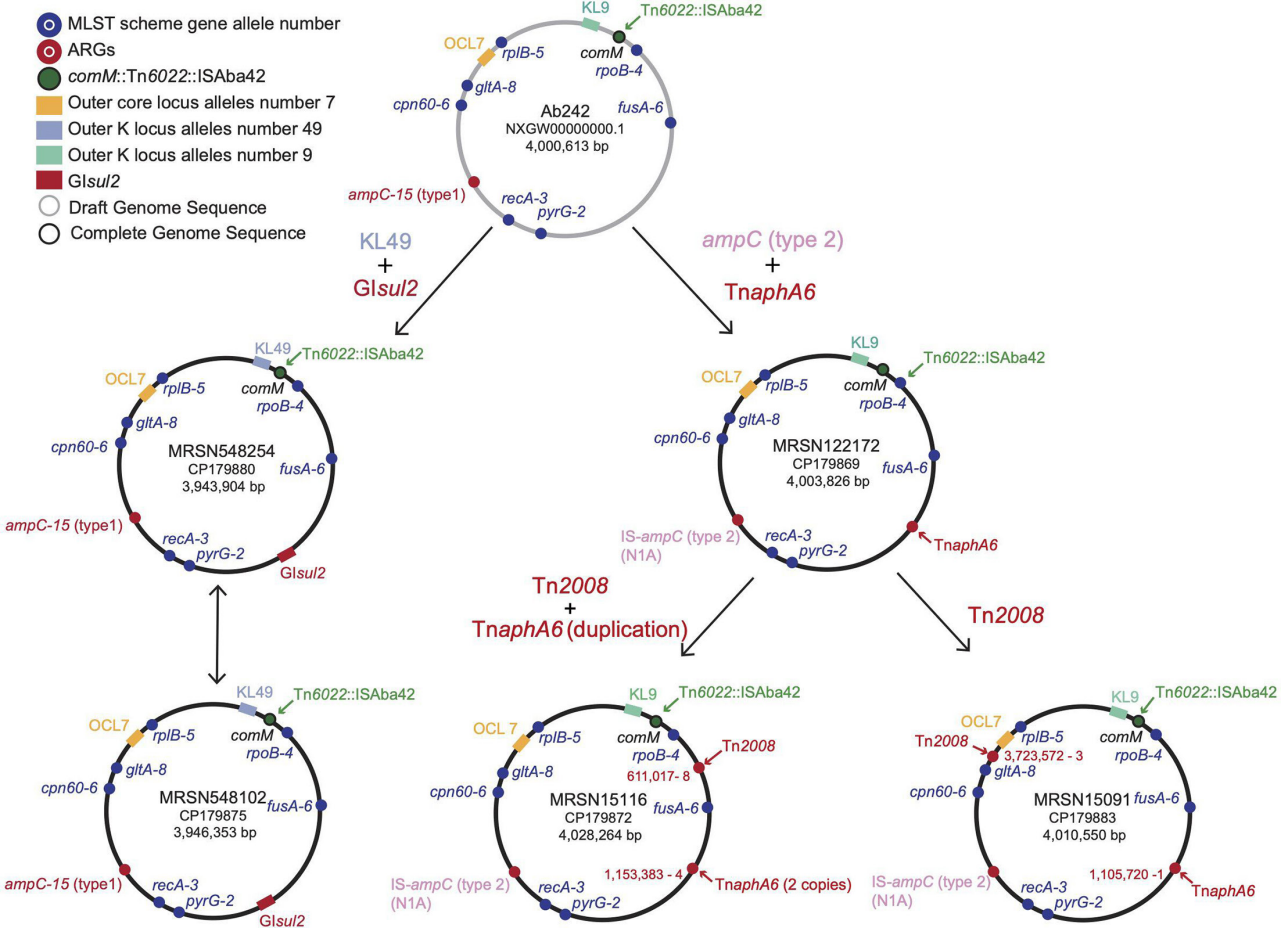
Properties of the five completed strains in this study. Each strain’s chromosomal layout is shown, with the MLST Pasteur scheme allele numbers mapped to their chromosomal locations. Dots indicate the positions of genes, while blocks highlight regions with multiple genes. The *comM* gene is disrupted by the integration of Tn*6022*, represented in the same colour. Arrows indicate insertion events at specific loci. Red highlights represent ARGs, while green represents transposons unrelated to resistance.

Together, several events have led to the current complexity in the genomic features of ST15. As an example, we further examined major genetic events that led to the diversification of genomes across C2 (MRSN548254, MRSN548102 and C4), C1.2 (Ab242) and C1.1.1.1a (MRSN15116, MRSN122172 and MRSN15091) (Figs2  3). These analyses suggest that Ab242 is more ancestral compared to the other genomes analysed (Fig. 3). It appears that an ancestral strain of Ab242 (representing outbreak strains in 1997 in Argentina) gave rise to MRSN548254 and MRSN548102 by several major events including a major sequence switch in the K locus (KL9 to KL49) and acquisition of GI*sul2* (via Tn*6174*; see above) (Fig. 3). MRSN122172 has arisen by acquisition of the Tn*aphA6* amikacin resistance transposon and a chromosomal replacement in the *ampC* region (replacing *ampC-15* with an ISAba1 activated *ampC* type 2; see below). Further evolution of MRSN122172, acquiring Tn*2008* and duplication of Tn*aphA6,* also led to the emergence of the carbapenem-resistant strain MRSN15116. However, Tn*aphA6* is not duplicated in MRSN15091, and Tn*2008* is also in a different chromosomal position, suggesting a different evolutionary path (Fig. 3). Here, we only looked at a few major events in a few complete genomes, but the complexity (of these features in their respective groups) indicates many intricate events leading to the successful spread of these strains.

### Evolution of the chromosomal *ampC* region via ISAba1 insertion and homologous r*e*combination

**Fig. 4.**
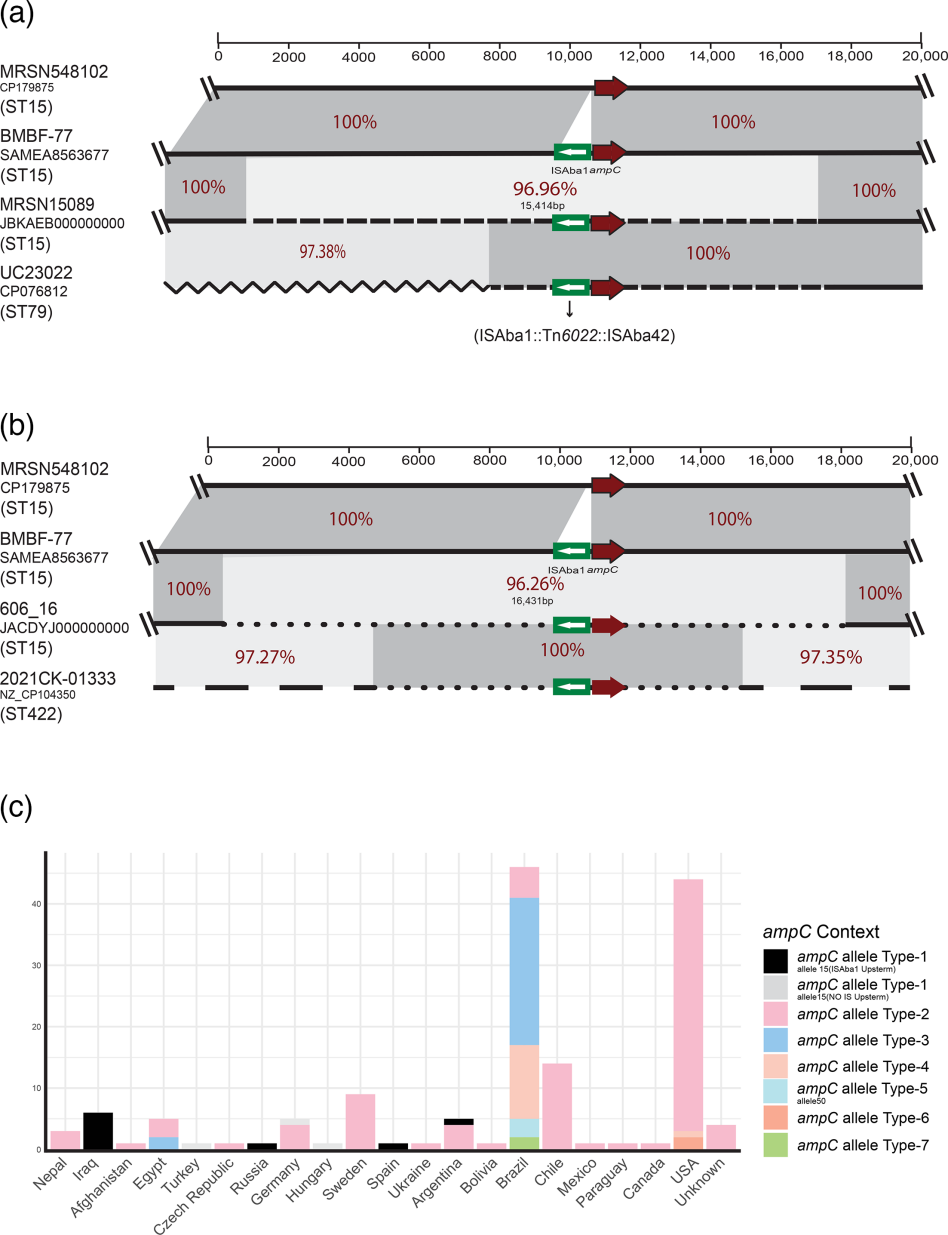
Schematic representation of the *ampC* chromosomal segments acquired by various STs. Horizontal arrows indicate the extent and orientation of genes, while thin horizontal lines delineate the boundaries of homologous recombination segments. The *ampC* gene is highlighted with red arrows, and green boxes represent insertion sequences. Regions with significant DNA identity are shaded in grey, with percentage identities displayed in red.

In *A. baumannii*, resistance to third-generation cephalosporins often occurs through the insertion of an IS, mainly ISAba1 and occasionally ISAba125, at a specific position upstream of the chromosomal *ampC* gene [39]. These IS provide the gene with a strong promoter, enhancing *ampC* expression and resulting in high levels of resistance to third-generation cephalosporins such as cefotaxime and ceftazidime [39]. In addition, we previously reported several examples where *A. baumannii* strains belonging to ST1 became resistant to third-generation cephalosporins through the acquisition of large chromosomal segments containing the ISAba1-*ampC* structure from a different strain [2,40]. Here, we analysed the evolution of the *ampC* region in the ST15 genomes and only found 12 genomes that appeared to contain the original ST15 *ampC* sequence (the *ampC*-15 allele). Amongst these, nine genomes (eight in the C2 clade and one in C1.2, Fig. 2) did not contain any IS upstream of the gene, indicating the original genetic structure. The remaining three genomes (all in C2) included a copy of ISAba1 located 9 bp upstream of *ampC*’s start codon, indicating direct insertion of the IS upstream of the original ST15 *ampC* allele (Fig. 4a, and Fig.4b). In addition, 7 distinct *ampC* alleles (named types 1–7) and 5 variants were found in the remaining 140 genomes, suggesting multiple acquisition events via homologous recombination (Table 2). The recombinant DNA segments ranged in size from ~15.4 kb (*ampC* sequence type 2, identified in 54 genomes) to just over 33 kb (including *ampC* sequence type 2c, found in two genomes) and shared DNA identities ranging from 95.89% to 96.96% compared to *ampC-15*. Type 2 was the most common, found in 52 genomes from diverse geographical regions (Fig. 4c). We tracked the recombinant *ampC* regions, and in all cases, novel types 4 and 5 (Table 2) found identical sequences in distinct clonal types. For example, types 1 and 2 were found in ST79 and ST422, indicating they are either likely sources or have also acquired these segments from another strain. Previously, we reported several cases where the ISAba1-*ampC* structure had been acquired as large DNA segments via homologous recombination in ST1 genomes [2,40]. Here, we report an extensive degree of exchange events in this region, leading to the acquisition of ISAba1-*ampC* in ST15s, suggesting an important role for homologous recombination in the acquisition of IS-activated *ampC* genes and that resistance gene acquisition via recombination may be more widespread than currently perceived. This is consistent with previous findings of Hernández-González IL. *et al*., which reported a significant role for resistance acquisition via recombination [41]. However, this requires a comprehensive assessment across different clonal types.

**Table 2.** *ampC* types and their possible sources identified in ST15 genomes

*ampC* sequence type	*ampC* allele	% identity(# of SNP)*	No. ofST15 strain	Length of recombinant*ampC* region†	Found in‡
1 (original)	15	100 (0)	12	–	ST15
2	Novel 1	96.96 (35)	52	15,414	ST97§
2a	Novel 1 a	96.96 (35)	20	19,606	ST79 and ST1§
2b	Novel 1b	96.87 (36)	20	15,405	ST79§
2c	Novel 1 c	96.87 (36)	2	33,886	ST109¶
3	Novel 2	96.26 (43)	13	16,431	ST422**
3a	Novel 2 a	96.18 (44)	3	16,409	ST422††
3c	Novel 2 c	96.18 (44)	10	21,172	ST422**
4	Novel 3	96.18 (44)	13	22,154	ST374 and ST1
5	50	96.18 (44)	2	21,173	ST79§
6	Novel 4	96.00 (46)	3	21,244	Not Known
7	Novel 5	95.89 (48)	2	21,049	Not Known

*Numbers indicate % DNA identity compared to the ST15 original *ampC* sequence with numbers in brackets representing the number of single base substitutions.

†Compared them to ST15 strain with original sequence (MRSN48102).

‡*ampC* batch possible source.

§Found this *ampC* sequence in some of the ST79 strains, but our analysis indicates that it is not the original sequence of ST79. These strains may have been acquired from another source.

¶The closest match to this sequence is ST109.

**The source of the *ampC* Novel 2 gene is ST422, with 16 strains listed on NCBI. For example, one of these strains is (e.g. in 2021CK-01333). Additionally, two other ST422 strains share the same *ampC* sequence as Novel 2 c.

††Differs by 2 bp compared to *ampC* in ST422 strains.

### Homologous recombination impacts diverse genomic regions

**Fig. 5.**
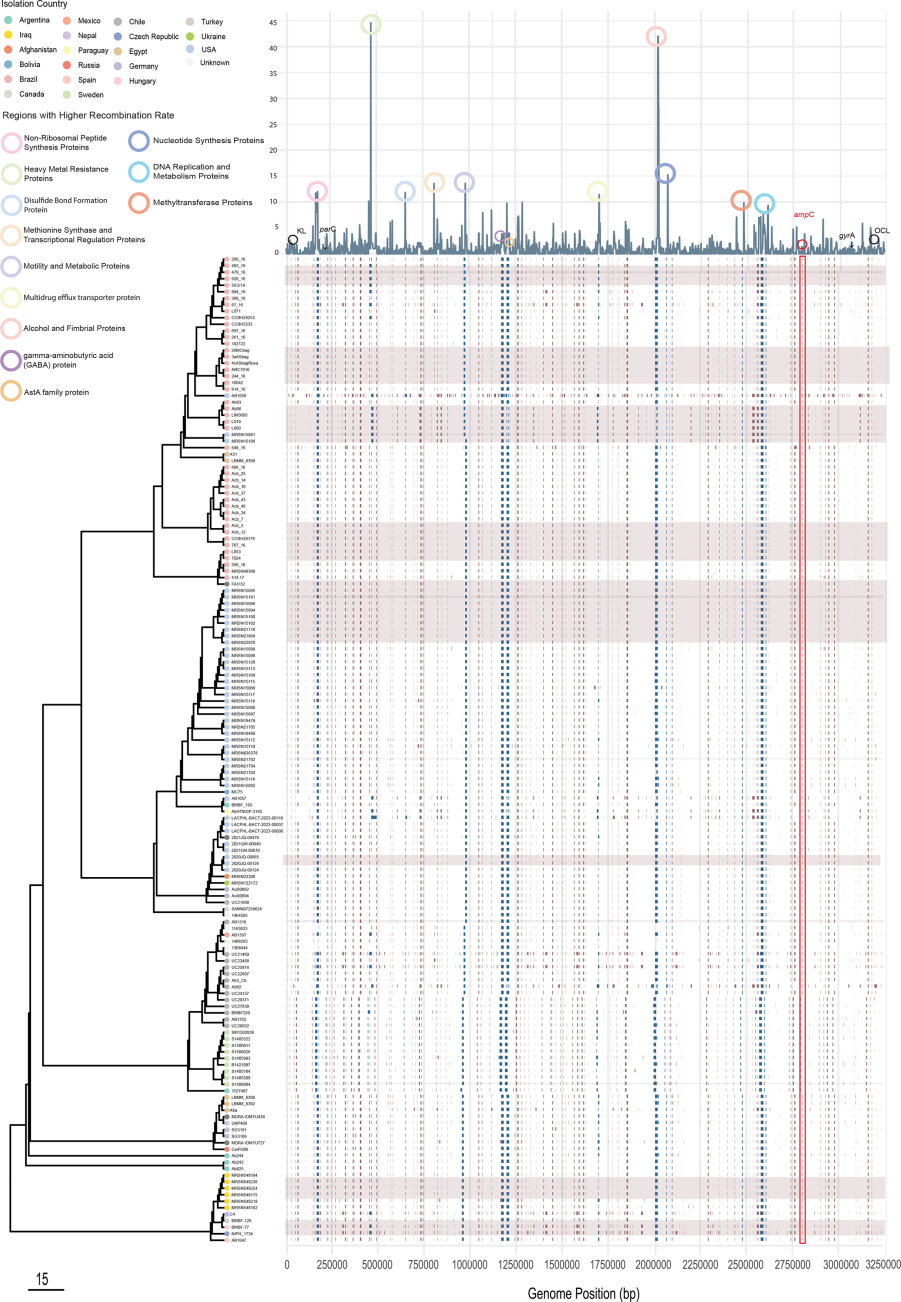
Phylogenetic tree and homologous recombination hotspots in ST15 strains of *A. baumannii*. The phylogenetic tree depicts relationships amongst strains, with homologous recombination patches shown as red blocks in the genomic alignment. Blue vertical lines highlight recombination hotspots, which correspond to peaks in the recombination plot and are associated with annotated loci. Key genomic features are annotated, including KL loci, OCL, *parC*, *gyrA* and *ampC* (highlighted in a red box). Tree nodes are colour-coded by country of origin, illustrating the geographical distribution of ST15 strains.

In addition to the *ampC* region, we identified chromosomal regions acquired via homologous recombination in ST15 genomes. It was found that across the ST15 set on average, 8.31% (total genome sizes 581,526,899 vs. the size of recombination blocks 48,313,096 identified by *Gubbins*; see ‘Methods’) of the genomes represent acquired sequences through homologous recombination. Notably, the recombinant regions were distributed across the chromosomes including genes involved in diverse functions, ranging from basic cell metabolism, regulatory functions, siderophores, surface polysaccharides, motility and indeed antimicrobial resistance (Fig. 5). Twelve regions with the highest recombination rates across the set are indicated in Fig. 5. Cells benefit from genetic exchanges in regions associated with resistance (i.e. by acquiring IS-activated *ampC* structures, Fig. 5) and in regions affecting surface polysaccharides, where K locus switching promotes immune evasion. However, the benefits of exchanges at other loci remain less clear and warrant further investigation.

### Plasmid *rep* distributions and acquisition of *bla*_CARB-50_ via plasmids carrying *dif *modules

It is now known that plasmids play an important role in the acquisition and spread of antibiotic resistance genes in *A. baumannii* [29,42]. We used our recently developed *Acinetobacter* plasmid typing scheme [29] to screen for plasmid replication (*rep*/Rep) sequences. R3-type Reps were found to be the most abundant type across the set with 143 genomes having one or more R3-type sequence, followed by R1-type (43 genomes) and RP-type (16 genomes) (Fig. 2). The most common R3-type sequences were R3-T14 (*n*=116), followed by R3-T60 (*n*=42) and R3-T3 (*n*=41). While the *rep* sequences were spread throughout the set, the top sub-clade of C1.1.1.1a was dominated by R3-type sequences, while in the bottom sub-clade (of C1.1.1.1a), the R3-T14-type was more abundant. Notably, this sub-clade did not include any R3-T60 sequences (Fig. 2). We previously showed that the R3-T3 group is exemplified by pABTJ2 (GenBank accession no. CP004359.1) and represents mostly cryptic plasmids of around 100 kb. Amongst these, we studied a set of R3-T14 plasmids as they carry the *bla*_CARB_ *β*-lactam resistance gene and were also present in our complete MRSN genomes (Table 1).

**Fig. 6.**
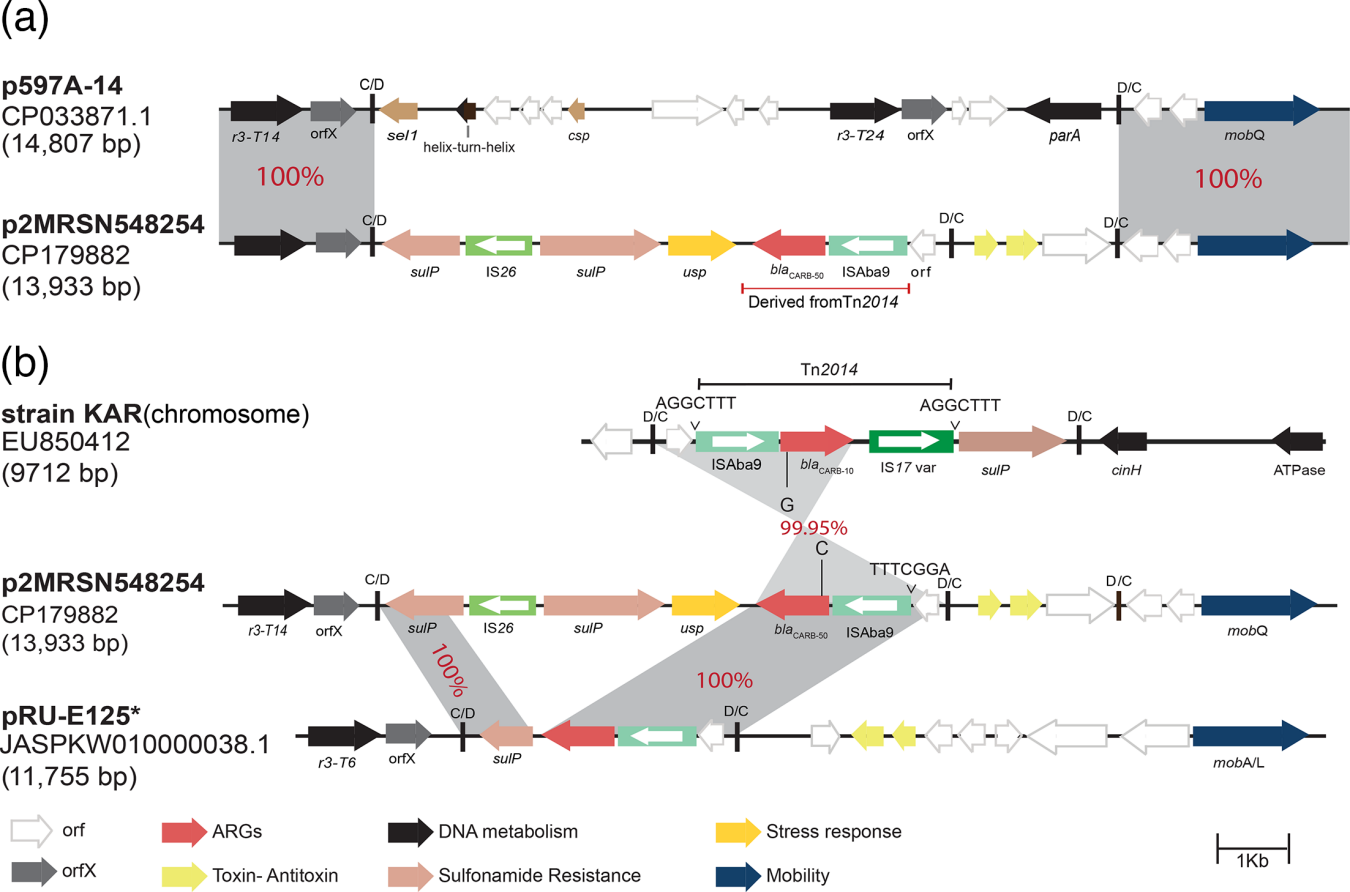
Schematic representation of the plasmid carrying *bla*_CARB-50_. The grey shading in both panels highlights shared genes, with percentage identities shown in red. ‘C/D’ and ‘D/C’ indicate *dif* modules. The *bla*_CARB_ gene is marked with a red arrow, while green boxes represent insertion sequences, with variations in green colour indicating different insertion sequences. The (*) shows the plasmid named here. The distinction between *bla*_CARB-50_ and *bla*_CARB-10_ is illustrated by the lines ‘C’ and ‘G’, highlighting a single nucleotide variation.

Variants of the *bla*_CARB_ gene encode carbenicillin-hydrolysing *β*-lactamases (also named CARB enzymes), which are narrow-spectrum class A penicillinases. CARB enzymes are divided into two subgroups, namely the CARB and RTG subgroups. CARB-10 belongs to the RTG-4 group and hydrolyses cefepime and cefpirome and weakly hydrolyses ceftazidime [43]. The *bla*_CARB-10_ gene has been shown in Tn*2014*, a 2,980 bp class one transposon made of a central segment, bounded by an ISAba9 and an IS*17* variant (Fig. 6). Several *A. baumannii* plasmids have recently been shown to carry and spread antibiotic resistance genes via *dif* modules (DNA segments flanked by binding sites for XerC and XerD recombinases) [1,42, 44,47]. Here, we found the *bla*_CARB_ gene in p2MRSN548254 (a 13 kb plasmid encoding R3-T14) in a remnant of Tn*2014,* which is embedded in a *dif* module (Fig. 2). A closely related plasmid, p597A-14 (GenBank accession no. CP033871), with a backbone (*rep* and *mob* regions) identical to p2MRSN548254, includes a completely different set of *dif* modules (Fig. 6a), indicating that the *bla*_CARB_ *dif* module is acquired via recombination. We further tracked Tn*2014* and found multiple variants, including a complete copy, again in a *dif* module, inserted in the chromosome of *A. baumannii* KAR (GenBank accession no. EU850412) (Fig. 6b). We also found the *bla*_CARB_ gene in an additional five MRSN strains in a similar context. To date, *A. baumannii dif* modules have been predominantly found in plasmids [1,29, 42, 44,46]; however, here, we report a chromosomal *dif* module, carrying *bla*_CARB_, indicating that these modules are not restricted to plasmids and that they can also target and reside on chromosomes.

**Fig. 7.**
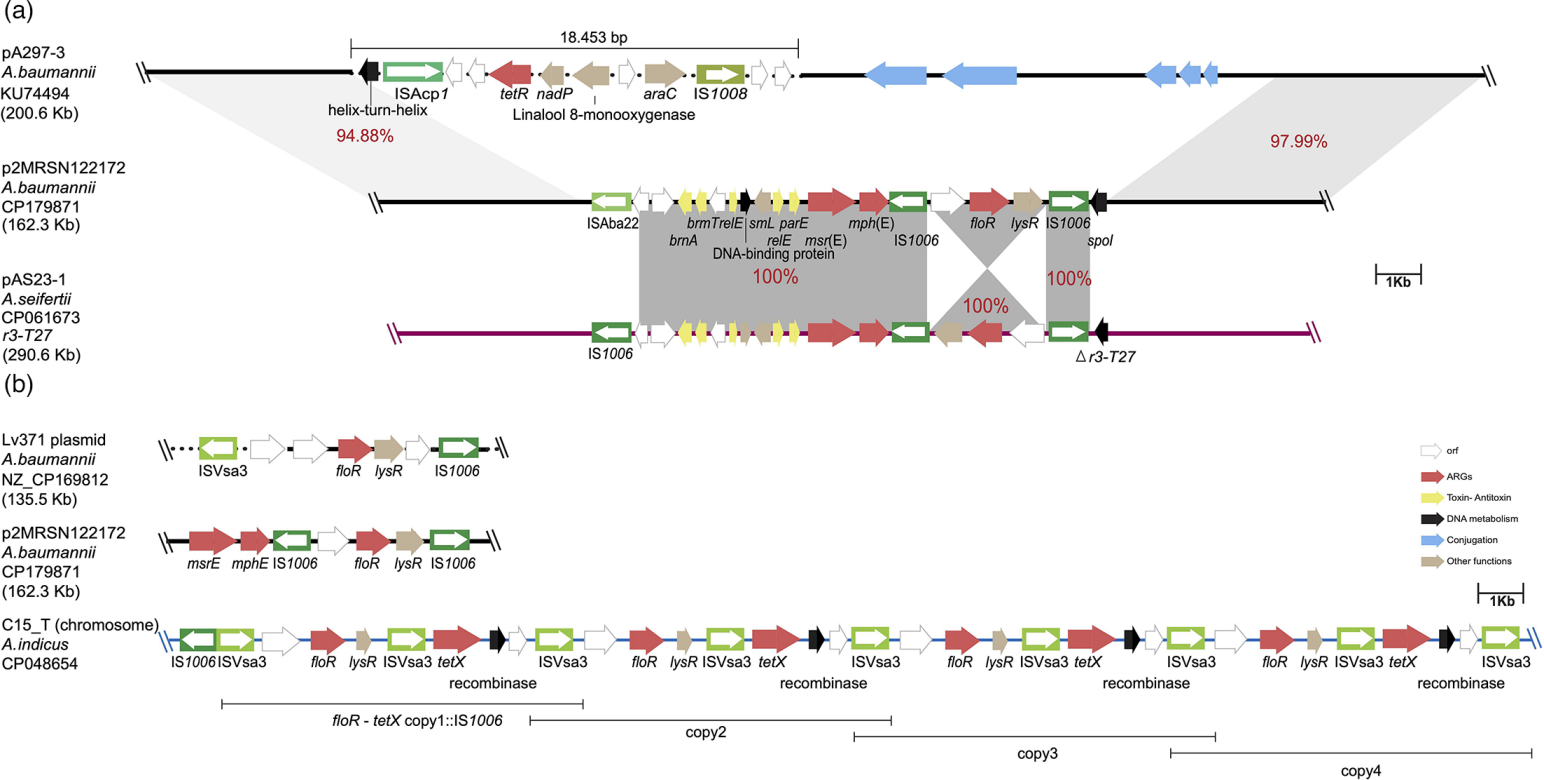
Structural overview of the plasmid harbouring *floR*, *msr*(E) and *mph*(E) genes. Grey-shaded regions indicate shared genetic content across plasmids, with percentage identities labelled in red. Antibiotic resistance genes are highlighted with red arrows, while green boxes indicate insertion sequences, with different greens representing variations. The boundary of the *floR-tetX* region is shown under the C15_T chromosome as lines and is repeated across four copies, as indicated by the labelled segments.

We also analysed the genetic context of the *msr*(E) and *mph*(E) macrolide and the *floR* chloramphenicol resistance genes in our complete plasmids. In p2MRSN122172, a representative of plasmids with *msr-mph*(E) and *floR,* these genes were in an ~16 kb region flanked by ISAba22 and IS*1006* (Fig. 7a). p2MRSN122172 belongs to the pA297-3 plasmid type [42,48], with no identifiable Rep. However, a closely related region in pAS23-1, an *Acinetobacter seifertii* plasmid encoding an R3-T29 Rep, also included this structure indicating that the *msr-mph*(E)-*floR* region can be exchanged between plasmids, presumably via IS*1006*. We also found the *msr-mph*(E)-*floR* region in several additional unrelated plasmids (e.g. pLv371, Fig. 7b) as well as a closely related structure with a *tetX* in the chromosome of an *Acinetobacter indicus* (strain C15_T; GenBank accession no. CP048654). Interestingly, in C15_T, the region between the two ISVsa3 copies was duplicated four times, presumably via the function of this IS. This phenomenon has previously been reported on different antibiotic resistance genes, including *aphA1* [49,50], *aphA6* [51] and *bla*_NDM_ [51]. This study adds *floR* and *tetX* to those previously reported and shows that ISVsa3 can also lead to tandem amplification of DNA segments that include resistance genes.

### Tracking the spread and origin

**Fig. 8.**
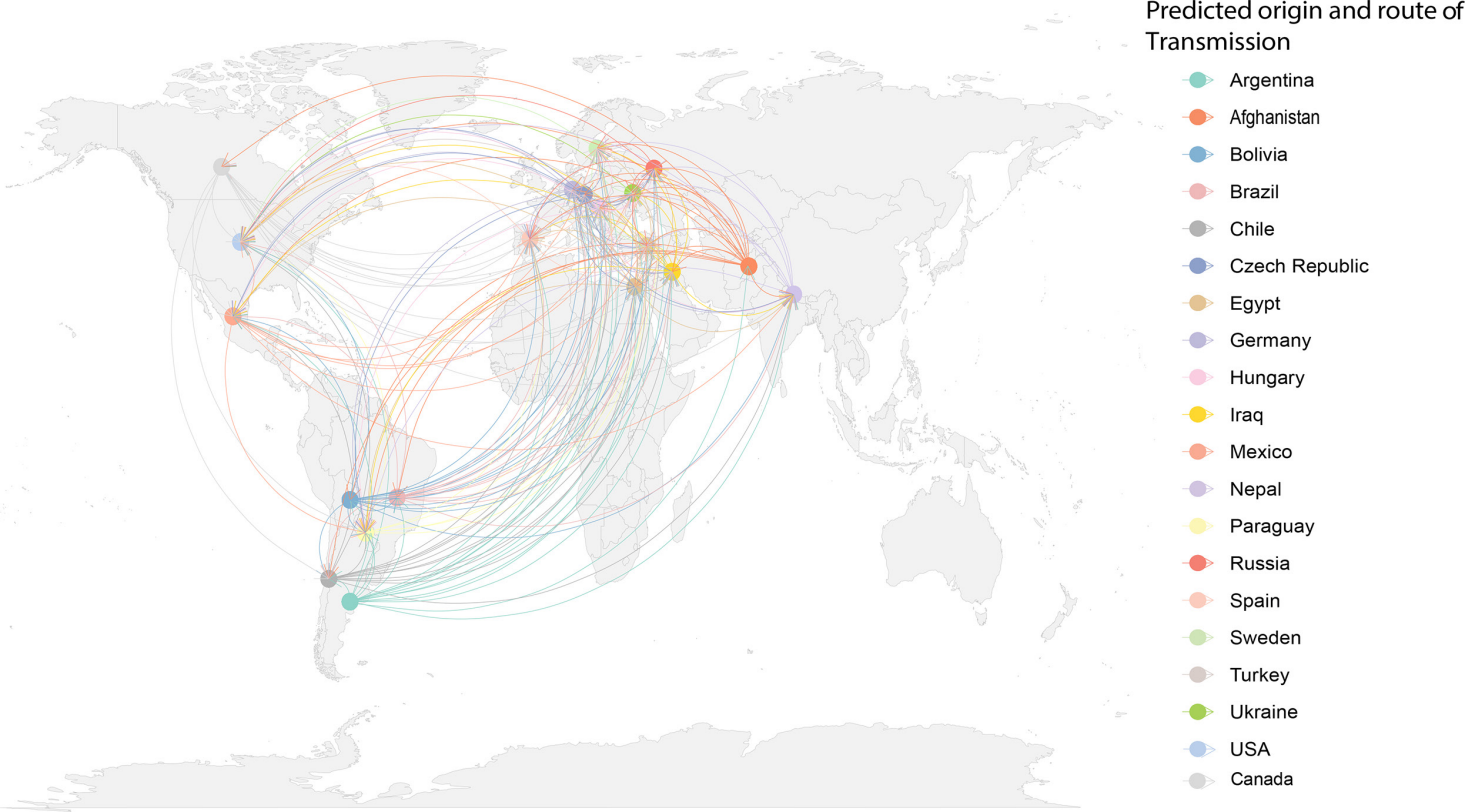
Global distribution and movement of *A. baumannii* ST15 strains, with Argentina identified as the predicted origin. Coloured lines represent the transmission pathways to various destination countries, with each line representing the ancestral links (of strains) to their common ancestor. The colour of the line indicates the predicted origin or transmission routes.

It has been shown that ST15 genomes belong to a distinct clade within the global phylogeny of *A. baumannii* genomes [12]. Given the global dissemination of ST15 as a drug-resistant clone, we investigated its transmission pathways to determine its origin using the *Patristic* program, as previously described [32]. *Patristic* computes the evolutionary distance between branches on a phylogenetic tree by summing the branch lengths along the shortest connecting path, thereby quantifying the cumulative genetic divergence since the last common ancestor. Our analysis revealed a complex transmission route. We identified countries, such as Argentina and Afghanistan, that acted as primary sample sources, whereas others, including the USA, Ukraine and Turkey, predominantly served as recipients. Our data suggest a significant directional flow of ST15, originating primarily from Argentina, indicating its role as the predicted origin of transmission, which is in alignment with early isolation data from this country (Fig. 8). This is consistent with the isolation of Argentinian strains in 1997 and the analysis of the accessory genomes and supports the hypothesis that the outbreak of ST15 MDR, carbapenem-resistant strains at the HECA in Rosario, Argentina [33], may represent the source. However, we also acknowledge that the discovery and sequencing of isolates predating 1997 from other countries (i.e. more ancestral than our earliest Argentine genomes) could modify this inference of Argentina being the source for ST15s. This remains to be shown. As additional genomes are sequenced, further clarity is expected regarding the evolutionary history of this significant clone.

## Conclusions

This study specifically addresses the understudied ST15 lineage, showing that they acquire and mobilize genes via unique recombination hotspots and MGEs, compared to other globally distributed clones, underscoring that resistance evolution is clonal-type specific. Indeed, our analysis revealed a complex evolutionary narrative underlying the emergence and global dissemination of this MDR clone. Our study demonstrates that extensive homologous recombination, coupled with the acquisition and mobilisation of key resistance genes via transposons and plasmids, has driven the diversification of ST15. Notably, our phylogenetic analyses identify distinct clades within ST15, each characterized by specific traits. We further suggest that Argentina may be the source of ST15 emergence, given the existing sequence data. Similar to other *A. baumannii* MDR clones, our findings show the capacity of ST15 to evolve through multiple genetic mechanisms to achieve resistance to various antibiotics, leading to its success and therefore global dissemination. Our results also indicate the need to broaden the scope of genomic surveillance efforts to include understudied but clinically relevant lineages such as ST15.

## Supplementary material

10.1099/mgen.0.001450 Table S1.

## Abbreviations

ARGs: antibiotic resistance genes
HECA: Hospital de Emergencias Clemente Alvarez
KL: capsule locus
MDR: multidrug-resistant
ML: maximum likelihood
MLST: multilocus sequence typing
MRSN: Multidrug-Resistant Organism Repository and Surveillance Network
NCBI: National Center for Biotechnology Information
OCL: outer core locus
STs: sequence types
